# 

**Published:** 1874-05

**Authors:** 


### Contents of May Number.

### ORIGINAL DEPARTMENT.

ART. I—Ingrowing of the Toe-Nail. By Prof. F. H. Hamilton, M. D. . 361
ART. II—Abstract of Proceedings of Buffalo Medical Association...... 363
ART. III—Albany County Medical Society. Semi-Monthly Meeting, March 11th, 1874........................................... 373

### MISCELLANEOUS.

Croton Chloral........................................................ 378
Defective Drainage........................................................ 381
Development of Intermittent Fever by Obstructed Drains.............. 382
Morbid Impulses........................................................ 383
Treatment of Vascular Naevi of Galvanic Cautery...................... 387
Ovariotomy under Difficulties........................................ 389
Neurasthenia and its Treatment........................................ 391

### EDITORIAL DEPARTMENT.

The Standard of Medical Education in America........................ 393
Medical Items........................................................ 395
Obituary........................................................ 396

BOOKS REVIEWED:
History of the American Ambulance in Paris.............. 397
The Puerperal Diseases. By Fordyce Barker.............. 398
Barnes on Diseases of Women.............................. 399
Books and Pamphlets Received........................................ 400

